# Electricity Generation and Wastewater Treatment of Oil Refinery in Microbial Fuel Cells Using *Pseudomonas putida*

**DOI:** 10.3390/ijms150916772

**Published:** 2014-09-22

**Authors:** Dip Majumder, Jyoti Prakash Maity, Min-Jen Tseng, Vanita Roshan Nimje, Hau-Ren Chen, Chien-Cheng Chen, Young-Fo Chang, Tsui-Chu Yang, Chen-Yen Chen

**Affiliations:** 1 Department of Life Science, National Chung Cheng University, 168 University Road, Minhsiung, Chia-Yi 62102, Taiwan; E-Mails: dipmajumder1@gmail.com (D.M.); biomjt@ccu.edu.tw (M.-J.T.); biohrc@ccu.edu.tw (H.-R.C.); 2 Department of Earth and Environmental Sciences, National Chung Cheng University 168, University Rd., Min-Hsiung, Chia-Yi 62102, Taiwan; E-Mails: jyoti_maity@yahoo.com (J.P.M.); seichyo@ccu.edu.tw (Y.-F.C.); 3 Department of Chemical Engineering, University Institute of Chemical Technology, Nathalal Parekh Road, Matunga East, Mumbai, Maharashtra 400019, India; E-Mail: vanita.nimje@gmail.com; 4 Department of Biotechnology, National Kaohsiung Normal University, No. 62, Shenjhong Rd., Yanchao Township, Kaohsiung County 82444, Taiwan; E-Mail: cheng@nknucc.nknu.edu.tw; 5 Department of Hotel and Restaurant Management, Chia-Nan University of Pharmacy and Science, Tainan 71751, Taiwan; E-Mail: t10344@mail.chna.edu.tw; 6 Advanced Institute of Manufacturing with High-tech Innovations, National Chung Cheng University, Minhsiung, Chiayi 62102, Taiwan

**Keywords:** microbial fuel cell, oil refinery, air-cathode, *Pseudomonasputida*, chemical oxygen demand

## Abstract

Microbial fuel cells (MFCs) represent a novel platform for treating wastewater and at the same time generating electricity. Using *Pseudomonas* *putida* (*BCRC 1059*), a wild-type bacterium, we demonstrated that the refinery wastewater could be treated and also generate electric current in an air-cathode chamber over four-batch cycles for 63 cumulative days. Our study indicated that the oil refinery wastewater containing 2213 mg/L (ppm) chemical oxygen demand (COD) could be used as a substrate for electricity generation in the reactor of the MFC. A maximum voltage of 355 mV was obtained with the highest power density of 0.005 mW/cm^2^ in the third cycle with a maximum current density of 0.015 mA/cm^2^ in regard to the external resistor of 1000 Ω. A maximum coulombic efficiency of 6 × 10^−2^% was obtained in the fourth cycle. The removal efficiency of the COD reached 30% as a function of time. Electron transfer mechanism was studied using cyclic voltammetry, which indicated the presence of a soluble electron shuttle in the reactor. Our study demonstrated that oil refinery wastewater could be used as a substrate for electricity generation.

## 1. Introduction

Energy requirements have been increasing exponentially worldwide. At present, global energy requirements are mostly dependent on fossil fuels, which will eventually lead to an exhaustion of limited fossil energy sources. Combustion of fossil fuels also has serious negative effects on the environment due to CO_2_ emissions, which could be the main reason for climate change. Increased global demand for finite oil and natural gas reserves, and energy security concerns have intensified the search for alternatives to fossil fuels [1]. Bioelectrochemical systems such as microbial fuel cells (MFCs) are devices that exploit the ability of exo-electrogenic microbes to respire through the transfer of electrons outside the cell [2]. MFCs have been shown to convert the energy in organic matter present in wastewaters into electrical current [3,4,5,6]. In comparison to conventional fuel cells the key advantages of biological fuel cells are the mild operating conditions such as ambient temperature and near neutral pH. It could allow essentially infinite applications of potential fuel. However, there is a scarcity of suitable electrocatalysts for oxidation [7]. The principle of MFCs is based on the fact that generation of electricity is one of the basic properties of microorganisms, as they transfer electrons from an oxidized electron donor to an electron acceptor at a higher electrochemical potential [7]. Exo-electrogenic bacteria are mostly employed in MFC because exo-electrogenic bacteria transfer electrons to the anode of a MFC either through direct contact via highly conductive nanowires or membrane-associated proteins [2,7,8], or by using soluble electron shuttles [9]. Bioelectrogenesis was first demonstrated in 1911 by Potter who used *Saccharomyces cerevisiae* and some other species of bacteria with a Pt electrode immersed in sterile medium in a battery-like setup, and the chemical action of their vital process was utilized to develop electrical energy. Electrical energy was generated due to disintegration of organic compounds by microorganisms; Potter reported that he had obtained a voltage ranging from 0.3 to 0.5 volts [10].

Over the last few years, MFCs have been the focus of increasing interest due to their sustainable approach towards wastewater treatment along with use as an alternative source for power generation [11,12]. Domestic wastewater was used for electricity generation in several MFC configurations [13,14,15,16] and a maximum power density of 204 mW/m^2^ was demonstrated [1]. Huang and Logan reported the effectiveness of electricity production with paper recycling plant wastewater with maximum power density reaching 672 mW/m^2^ [17]. Beer brewery wastewater treatment using an air-cathode MFC was investigated by Feng and Wang [18,19] and a maximum power density of 528 mW/m^2^ was achieved [18]. In 2006, Krishnan *et al*. reported electrolytic treatment of beer brewery wastewater on the basis of *in-situ* hypochlorous acid generation, and a maximum current density of 74.5 mA/cm^2^ was achieved [20]. Starch processing wastewater was reported as being used for power generation using an air-cathode MFC with a maximum power density of 239.4 mW/m^2^ and a current density of 893.3 mA/m^2^ [21]. Swine wastewater treatment using a single chamber air-cathode MFC was studied and maximum power density of 261 mW/m^2^ was achieved [22]. Removal of odor from swine wastewater was investigated by Kim *et al*., and the maximum power density achieved was 228 mW/m^2^ [23]. Chocolate industry wastewater treatment using a double chamber MFC was studied by Patil and colleagues and the maximum current achieved was 3.02 and 2.3 A/m^2^ using a membrane and salt bridge respectively [6]. A single chamber MFC with an air-cathode was successfully created using a glucose-penicillin mixture or only penicillin as the fuel and a maximum current density of 10.73 A/m^2^ was achieved for penicillin and a maximum power density of 101.2 W/m^3^ was achieved for glucose-penicillin mixtures [24]. A MFC was employed to dispose of Cr^6+^ in real electroplating wastewater and the maximum power density of 1.6 W/m^2^ was generated at a coulombic efficiency of 12% [25].

So far, very few studies have been performed using oil refinery wastewater in MFC. Oil refinery wastewater is one of the major environmental pollutants if the treatment is not proper. It causes much environmental damage such as the abolition of microflora and fauna in water bodies that could spoil water quality. Most importantly it causes abnormal changes in the water eco-system. The major problem with oil refinery wastewater is the vast chemical oxygen demand (COD). We are reporting here the result of reduced COD using *Pseudomonas* *putida* (BCRC 1059) for the treatment of oil refinery wastewater in a single chamber air-cathode MFC.

## 2. Results and Discussion

### 2.1. Current Generation from Refinery Wastewater

**Figure 1:**
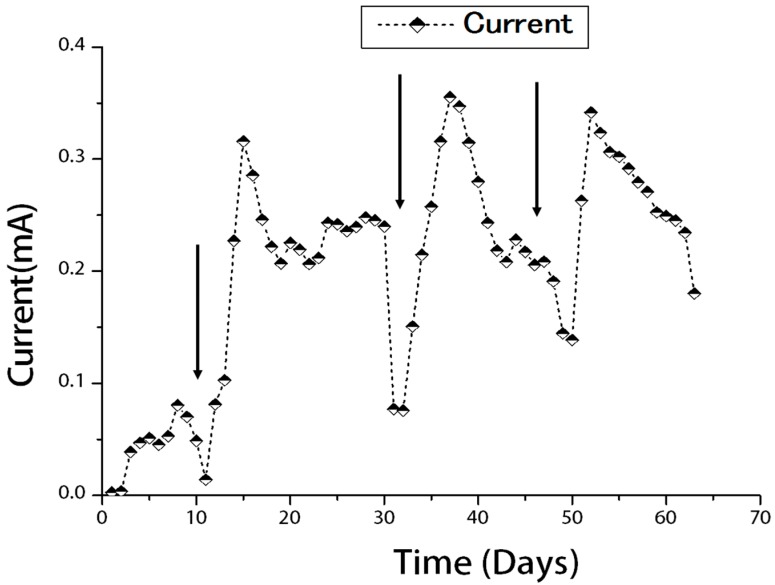
Current generation in air-cathode MFC for four successive batch cycles. Those arrows indicate the substrate inoculum addition as an end of each cycle.

The power generation shown in Figure 1 was observed over a period of four batch cycles with a fixed external resistance (1000 Ω). During the startup stage, there is a lag period of two days followed by inoculation and the course lasted for a total of 63 days from the beginning of the first cycle. An initial peak current of 0.08 mA was achieved in the first cycle. The bacterial population was restored by new substrate inoculation at the beginning of the second cycle and there was an immediate power generation of 81.26 mV. This result could be due to the difference in potential between the two electrodes based on both chemical and biological factors.

Thereafter a sharp increase in current was observed to reach 0.31 mA, which might indicate the bio-electrochemical activity of the microorganisms that then gradually started to decrease after 15 days. The third and the fourth cycles were performed as per the second cycle. As shown in Figure 1, greater current output was observed in the later feed-batch cycles, *i.e.*, third and fourth feed batches as compared with the first batch. Each cycle can be divided into three phases—ascending, stationary and declining. From Figure 1 it was observed that the stationary phase was longest in the second and third cycles. It is probably due to the formation of a biofilm seen in the reactor by electrochemically-active bacteria and the successful degradation of organic matter. The ascending phase was longer in the first cycle than in any other cycles owing to functioning of the biofilm. In the fourth cycle, however there was a sharp decrease from the stationary phase to the declining phase, which could be the inhibition of electron transfer from the bacteria to the anode surface by the matured bacterial biofilm.

### 2.2. Characterization of Microbial Fuel Cells (MFC)

**Figure 2:**
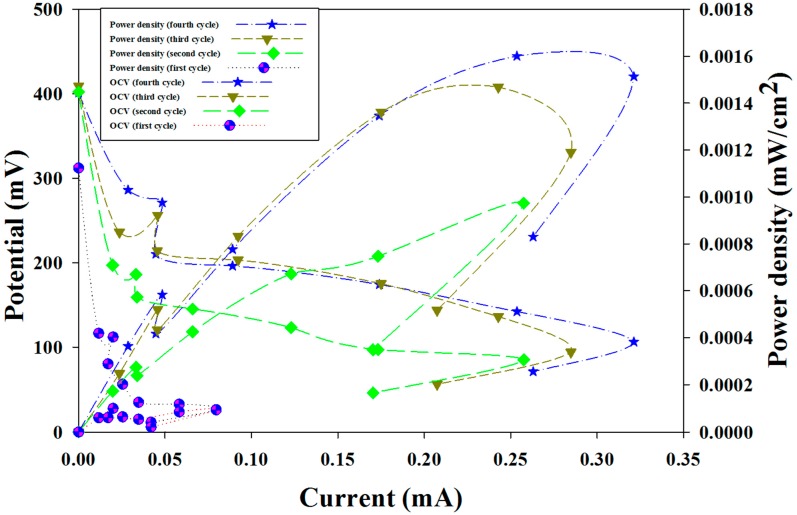
Polarization curve for four cycles.

One of the most important parameters of the MFC is the polarization curve, which is used to assess performance on the basis of current generation. A polarization curve represents voltage as a function of current. In a single batch cycle, the MFC was stabilized at the maximum steady voltage, and the power density and polarization curves were measured at several points by changing the external resistance from 0.27 to 10 kΩ. Figure 2 shows the curves of voltage, current and power density, which followed a similar pattern for all four cycles. Allocations of open circuit voltage (OCV), maximum power density, and maximum current output are presented in Table 1. According to Table 1, the OCVs of the four cycles were consecutively at 312 mV for the first cycle and 402 mV for the second cycle, 409 mV for third cycle and 401 mV for fourth cycle. In the status of open circuit, no current is circuited through the circuit and hence power production is null. There are three phases in the polarization curve of Figure 2: Activation losses, ohmic losses and mass transport limitation [1,2]. At the beginning of power production there is a drop of voltage, which could be attributed to activation loss of substrate diffusion. This finding is in agreement with results in the literature [1,2,26]. Similarly, a decrement of the current has been observed as the resistance increased, which is in agreement with the literature [1,2,12,26]. During the second phase of ohmic losses the current rose, and a linear relationship of voltage and current was exhibited. This is because of the resistance of electron and ion movement. This region formed an overshoot except during the first cycle, and hence reached their maximum power densities. The highest power density achieved was 0.0011 and 0.0015 mW/cm^2^ respectively for the third and fourth cycle, and the associated currents were 0.25 and 0.28 mA (Table 1). The third cycle and fourth cycle produced better power curves than the two initial ones. There is a steep drop of power density near to the maximum cell voltage, which could be attributed to mass transport losses respective of all cycles (Figure 2). According to the polarization curve, the optimum resistance for our study was 1000 Ω, which applies to all cycles.

**Table 1:** Different polarization and current output values for four fed batch cycles during operation with different external resistance (0.27–10 kΩ).

Cycle Number	Open Circuit Potential (mV)	Maximum Current (mA)	Maximum Power Density (mW/cm^2^)
Cycle 1	312	0.079612121	0.000092
Cycle 2	402	0.257575758	0.000973
Cycle 3	409	0.284848485	0.00119
Cycle 4	401	0.321212121	0.0015132

### 2.3. Cyclic Voltammetry

**Figure 3:**
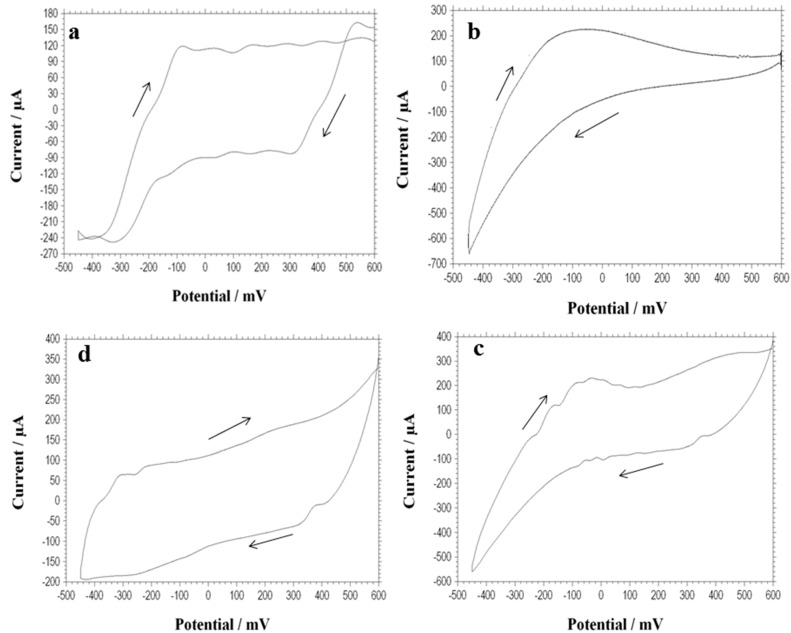
Cyclic voltammogram was recorded at a scan rate of 0.1 V/s for four batch cycles. Cycles are shown here as a clockwise direction (**a**: first cycle; **b**: second cycle; **c**: third cycle; and **d**: fourth cycle).

Cyclic voltammetry (CV) is one of the most familiar and versatile techniques used to reflect electrochemical reactions, which allow probing of the mechanics of redox and transport properties of a system in solution. It also enables measurement of redox activities between the components involved in a biochemical system and components bound to the bacteria. The voltammetry profiles (Figure 3) for the four batch cycles revealed there were noticeable variations in the electron discharge and energy generation patterns in the individual cycles. During the highest output of current, a voltammogram was recorded *in situ* with a scan rate of 0.1 Vs^−1^. From the CV profile a significant peak was found in the second and third cycles but not in the fourth cycle, in both the forward and reverse scans (Figure 3b–d). A small peak in the first cycle was also found (Figure 3a). In the 1st cycle, the oxidation peak of 0.12 mA was found at −100 mV (*vs.* Ag/AgCl) and a corresponding reduction peak was found near 0.1 mA (Figure 3a). For the second cycle an oxidation peak of 0.2 mA was found at −200 mV (*vs.* Ag/AgCl) and the reduction peak was shown at the same voltage (Figure 3b). For the third cycle, an oxidation peak of 0.21 mA was detected at −110 mV (*vs.* Ag/AgCl) and a corresponding reduction peak of −0.13 mA was detected at almost 30 mV (Figure 3c). However, for the fourth cycle the oxidative peak of 0.05 mA was very low at nearly −320 mV (*vs.* Ag/AgCl), and a reduction peak of 0.15 mA was detected (Figure 3d).

It is well known that the mechanism of anodic bacterial electron transfer is governed by three different mechanisms. One is the direct electron transfer between the electrode surface and bacterial membrane. Second is the mediated electron transfer which uses a redox active compound for the shuttle of the electron between the electrode and bacteria. The third one is wire electron transfer, which uses facilitated nanowire by bacteria for the transfer of electron to electrode [7]. In the case of the low current in first cycle, the biofilm was immature and was hence considered not to contribute much to the electron transfer (Figure 1 and Figure 3a). A strong oxidative peak was detected in both the second and third cycles, especially in the third cycle which indicated the biofilm could develop and mature after a long incubation time (Figure 1 and Figure 3b,c).

The strong oxidation/reduction peaks in the voltammogram have been found in the second and third cycles (Figure 3b,c), which implied that the studied bacteria became more electrochemically active. The active bacteria could produce redox active compounds that facilitate electron transfer. The production of current would rely on the availability of such compounds. Those profound concentrations of redox mediators could improve the electron transfer and hence generate more current. However, there was a fall in current in the fourth cycle, which reveals that the biofilm might become fully developed and aged. It does not play a key role in electron transfer but acts as an inhibitor for the block of the electrochemical property (Figure 3d).

It could be concluded that the observed peaks in voltammogram were due to redox active complexes produced by bacteria, which could control the electron transfer process for power generation. These redox active compounds might be attributed to the degradation of the substrate from refinery wastewater. The proficiency of the MFC reactor is dependent on the availability of the degraded derivatives.

### 2.4. Coulombic Efficiency as a Function of Power Density Using Refinery Wastewater

**Figure 4:**
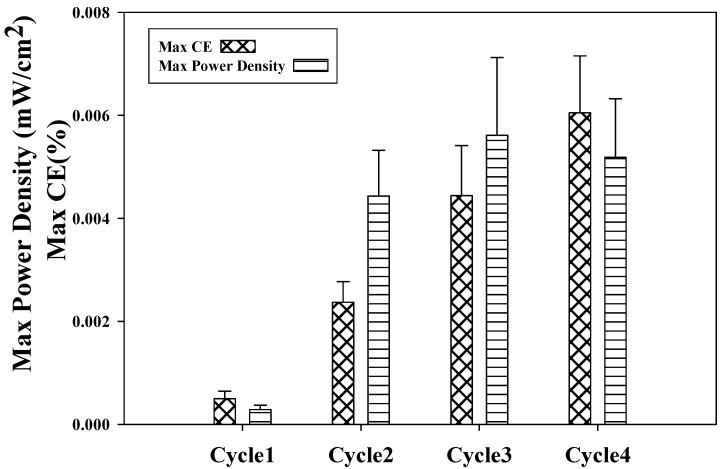
Coulombic efficiency as a function of power density using refinery wastewater for fourth batch cycles.

Coulombic efficiency (CE) and power density are the two major parameters used to describe and understand the phenomena that take place in the reactor of MFC. The data of Figure 4 showed that CE has been increasing with the increase of cycles. It ranged from 1 × 10^−3^% to 6 × 10^−3^% with a maximum of 6 × 10^−3^% for the fourth cycle. Maximum power densities were proportional to the changes in CE. This result is consistent with the result of Lu *et al*. [21]. However, maximum power density in the fourth cycle was found to decrease compared with the third cycle. This variation agreed with the result of CV and could be due to biological interference of biofilm aging. The low CE is a general issue in MFCs using real wastewater. Several probable factors including reactor configuration that could contribute to lower CE in MFCs were reported [19]. The other is the intake of electrons for other bacterial metabolic activities such as methanogenesis and fermentation [19].

In contradiction to the literature, our study did not reach the same maximum CE. The probable cause is the existence of additional existing electron acceptors such as nitrate and sulfate in oil refinery wastewater, which consumes electrons and thus lowers the CE [21]. This causes the additional flow of electrons from substrates to different acceptors. The dissipation of electrons hampered the gain of the highest CE. Oxygen diffusion through the cathode also accounts for loss of carbon compounds to aerobic respiration and degradation, resulting in a low CE [8]. Higher recovery of electrons represents more effective organic oxidization and lower loss of cells contributing to production of electricity. The highest CE achieved in this study, was not as high as compared with that achieved using other wastewater. This is probably because of the presence of other electron acceptors in the wastewater and oxygen diffusion during the batch process. Reactor configuration also could be improved to achieve high CE.

### 2.5. Chemical Oxygen Demand (COD) Removal and Wastewater Treatment Efficiency

**Figure 5:**
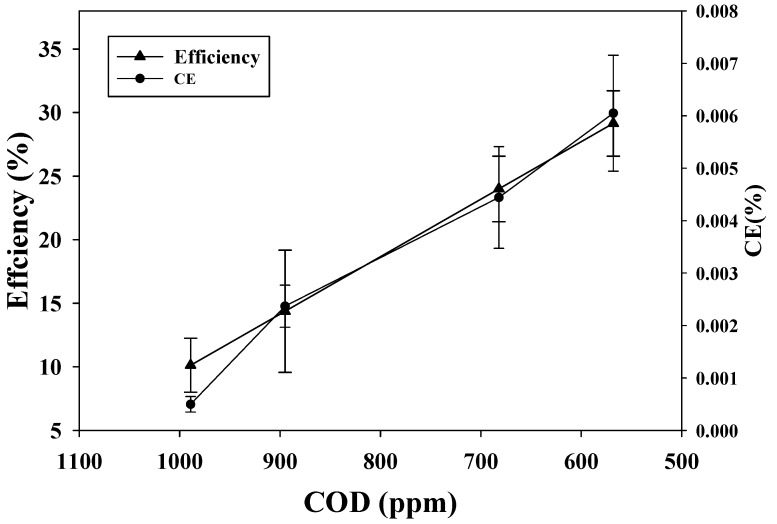
Refinery wastewater chemical oxygen demand (COD) removal efficiency and coulombic efficiency (CE) was obtained for four batch cycles.

One of the major goals of the MFC system is the treatment of wastewater by removing the load of COD. COD removal efficiency and coulombic efficiency was computed for each cycle as shown in Figure 5. The data showed that there is a proportional relationship between COD removal and CE. Initially, the COD removal efficiency was very low, as was the CE of the system, but a significant increase in both parameters was found as the cycle proceeded. COD removal was increased by higher CE. The CE could be increased in future studies by adding two layers of cloth to the cathode surface as shown by Fan *et al.* [27].

**Figure 6:**
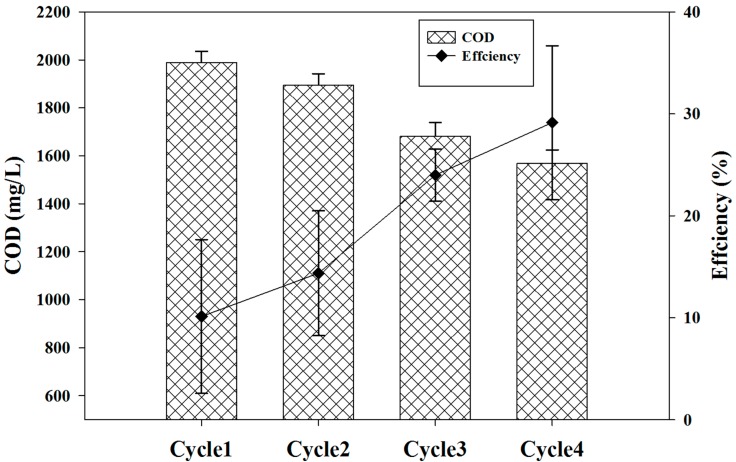
COD and removal efficiency was computed for four batch cycles of this study.

**Figure 7:**
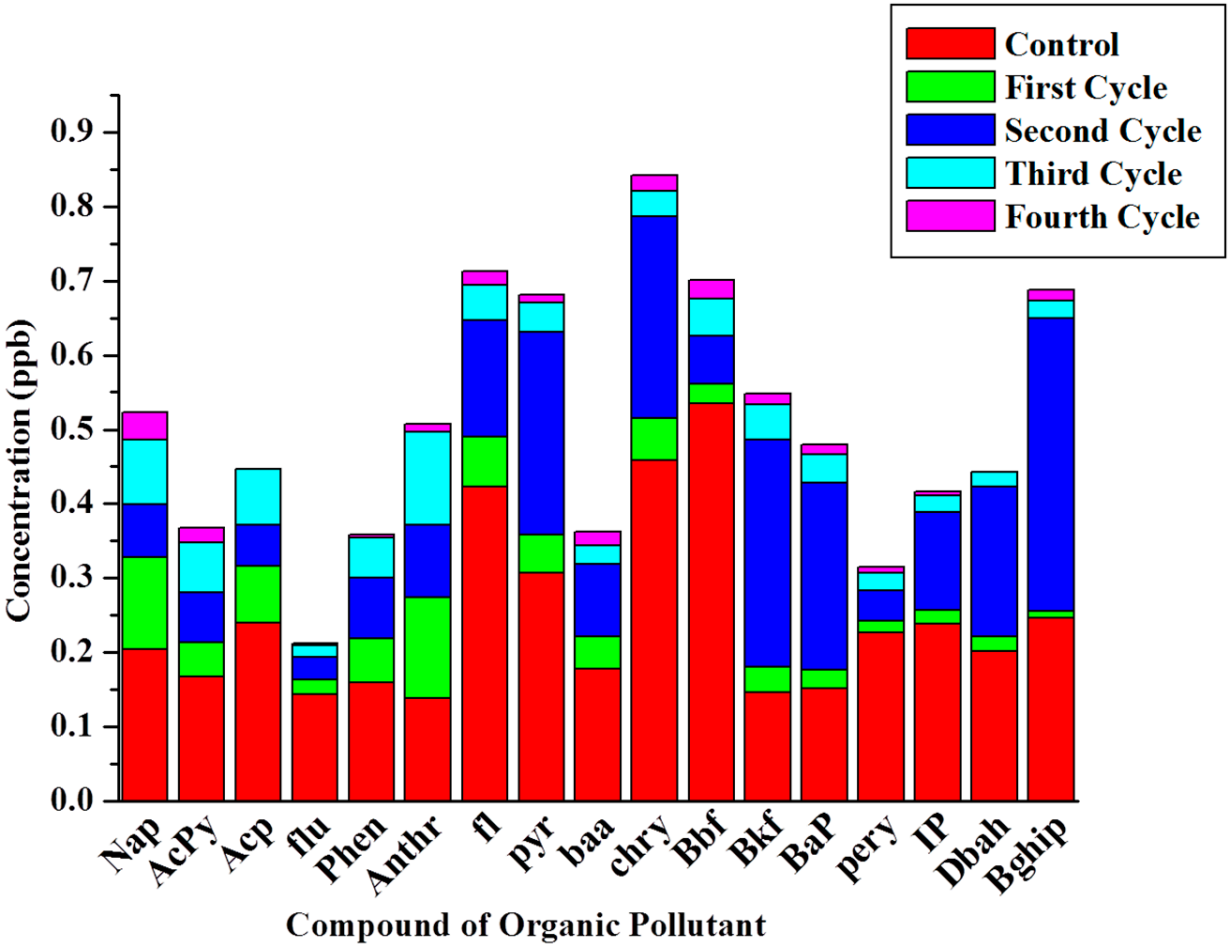
Degradation pattern of organic pollutants in refinery waste water.

The function of the MFC is to produce electricity as a result of the degradation of organic matter in wastewater. In our study, a MFC was developed that was able to perform electricity generation and wastewater treatment, simultaneously. The effluent COD concentrations and removal efficiencies for all of the four cycles are shown in Figure 6. The data showed that the COD removal efficiency increased as a function of time, ranging from 10.0% to 30.0%. Degradation patterns for petrochemical fractions for four batch cycles have been provided in Figure 7. The high COD removal was attributed to the long operation period in this study, which also extended the time for oxygen diffusion into the system, as a consequence of low CE. A COD removal efficiency of 30.0% and the low corresponding CE of 6 × 10^−3^% indicated that the major consumption of soluble and insoluble organic matter in refinery wastewater was not associated with power generation. Oxygen diffusion is not the only reason for this result and other factors such as other electron acceptors, biomass production and fermentation also need to be included. The wastewater treatment efficiency achieved is not sufficient as compared with other studies. We propose that the longer operation time is needed because the efficiency was found to increase as a function of time.

Substrate degradation is one of the main factors that lead to high current. Substrate degradation in this study did not achieve high capacity according to the efficiency of COD removal. The enhancement of substrate degradation by other methods is needed. It is also necessary to improve the electron transfer procedure between substrates and electrodes. The respective electrodes need to be rearranged. The improvement of reactor architecture needs to be taken into consideration. In addition, the use of a proper separator for the air cathode reactor as per Sevda *et* *al.*, led to increased COD removal [28]. In that study 77.56% of COD was removed using a Zirfon^®^ membrane compared with the 51.92% of removal using a Fumasep^®^ one.

The degradation of the petrochemical fraction at the beginning and at the end of each cycle was performed. The Figure 7 shows the degradation pattern for four cycles and the Table 2 shows the list of all poly-aromatic contaminants. The data shows the presence of aromatic fractions which has been found more often than aliphatic fractions in refinery waste-water after degradation. The electroactive bacteria are solely responsible for the degradation of organic pollutants with long incubation times. The spatial barrier of biofilm for electron transfer could be the reason for the reduction of current production in the fourth cycle.

**Table 2:** Details of the poly-aromatic hydrocarbons.

Abbreviation	Full Name	Formula	Molecular Weight
Nap	Naphthalene	C_10_H_8_	128
Acpy	Acenaphthylene	C_12_H_8_	152
Acp	Acenaphthene	C_12_H_10_	154
Flu	Fluorene	C_13_H_10_	166
Phen	Phenanthrene	C_14_H_10_	178
Anthr	Anthracene	C_14_H_10_	178
Fl	Fluoranthene	C_16_H_10_	202
Pyr	Pyrene	C_16_H_12_	202
Baa	Benzo[a]anthracene	C_18_H_12_	228
Chry	Chrysene	C_18_H_12_	228
Bbf	Benzo[b]fluoranthene	C_20_H_12_	252
Bkf	Benzo[k]fluoranthene	C_20_H_12_	252
Bap	Benzo[a]pyrene	C_20_H_12_	252
Pery	Perylene	C_20_H_12_	252
IP	Ideno[1,2,3-c,d]pyrene	C_22_H_12_	276
Dbah	Dibenzo[a,h]anthracene	C_22_H_14_	278
Bghip	Benzo[g,h,i] perylene	C_22_H_12_	276

## 3. Experimental Section

### 3.1. Oil Refinery Wastewater

Oil refinery wastewater was collected from CPC Corporation Chia-Yi, Taiwan and stored at 4 °C. The wastewater was diluted with the same volume of Milli-Q water before use and its COD was 2213 ppm. COD has been measured using a COD kit (COD Reagent; COD-S; CAS [7664–93–9], Merck, Taipei, Taiwan) and performed as per instruction.

### 3.2. Microorganisms and Medium

The biocatalyst *Pseudomonas putida* (BCRC 1059), a wild type bacterium, was used for the production of electricity from oil refinery wastewater. *P.* *putida* was cultured in a conical flask with 250 mL nutrient broth media and then incubated at temperatures of 35 ± 2 °C [29]. The nutrient broth media contained the following (gram/liter) peptic digest of animal tissue: 5, sodium chloride: 5, beef extract: 1.5, yeast extract: 1.5. The bacteria were cultured for 48–60 h, and were then inoculated into the reactor.

### 3.3. MFC Configuration

The MFC was comprised of a single chamber air-cathode fitted with a sidewise glass bridge and constructed from a 500 mL spherical glass-bottle with a three-electrode system. Both (cathode and anode) of the electrodes were prepared using carbon material and the cathode was polished with 40% PTFE (Polytetrafluoroethylene) to inhibit oxygen diffusion [30]. The anode electrode was a 3 × 3 cm^2^ piece of plain carbon cloth, with a projected area of 18 cm^2^, and the cathode was 4 × 3 cm^2^ with carbon cloth. Copper wire was used to connect the circuit, and the fuel cell was placed under a constant load by connecting the anode and cathode to an external resistance of 1000 Ω. The time periods of fed-batch mode process differed from substrate to substrate, conditional to the adjustment of the system; repetitive cycles were measured as per substrate availability. All experiments were conducted at room temperature.

### 3.4. Attainment of Data and Electrochemical Calculation

The voltage (*V*) across a resistor (*R*) was measured by a Pico Log recorder using Pico Log_v-5.09.4 recorder software (Pico Technology, Cambridgeshire, UK) with an RS232 interface connected to an ADC 20–21 A–D converter (Pico Technology). Data were recorded on a personal computer at 1 min intervals. The current (*I*) was determined by Ohm’s law (*I* = *V*/*R*) and the power density of the MFC was calculated as *P* = *IV*/*A*, where A was the anode surface. To obtain the polarization curves, different external loads were applied for a complete batch cycle, with a variable resistance from 10 to 0.27 kΩ. The MFC was then operated under a single constant external resistance (*R* = 1 kΩ), and the current was measured with respect to time.

The Coulombic efficiency was calculated as:

(1) $$\text{CE}=\frac{\int^{\text{​}}I\text{dt}}{\frac{\Delta \text{COD}}{32\times 1000}\times 4\times V\times 96480}\times 100\%$$

where *I* is the current, ∆COD is the difference in the COD value between the initial and final of the anode chamber, *V* is the volume of wastewater treated, 4 is the number of mol of 1 mol O_2_-related electrons, and 32 is the molecular weight of O_2_ [31].

### 3.5. Cyclic Voltammetry

Cyclic voltammetry was accomplished using a potentiostat (CHI 627C; CH Instruments Inc., Austin, TX, USA) connected to a computer-based CHI627C Electrochemical Analyzer in order to characterize the oxidation of organic material in wastewater at the biofilm anode surface. A scan rate of 0.1 Vs^−1^ was applied, ranging from −450 to 900 mV, to scan the biofilm anode. Cyclic voltammetry was performed using a well-known three-electrode system (CHI 627C; CH Instruments Inc., Austin, TX, USA), with the anode as the working electrode, an Ag/AgCl reference electrode, and a platinum wire as the counter electrode. The assembly was arranged such that contact between any of the electrodes was avoided.

## 4. Conclusions

Our study demonstrated that treatment of oil refinery wastewater could simultaneously generate electricity in an air-cathode MFC. The maximum voltage output of 355 mV was reached with the highest power density of 0.005 mW/cm^2^ in the third cycle. A maximum current density of 0.015 mA/cm^2^ was reached in the third cycle and the maximum coulombic efficiency (6 × 10^−2^) was observed in the fourth cycle, mainly caused by other electron acceptors in refinery wastewater and oxygen diffusion during the long operation period. A COD removal rate up to 30% was achieved. The combination with some well-known strains in MFC such as *Shewanella* and *Acinetobacter* would be the future prospects to achieve maximum power density and maximum COD removal in refinery waste water. Bacterial population dynamics can be detected and monitored in this design using the molecular biotechnology technique of RT-PCR to understand the interactions of enriched bacterial communities.
